# The relationship between living arrangements and depression among older adults in Shandong, China: The mediating role of social support

**DOI:** 10.3389/fpsyt.2022.896938

**Published:** 2022-11-14

**Authors:** Zhongfei Pei, Fangfang Hu, Wenzhe Qin, Yan Zhao, Xiaohong Zhang, Xinxia Cong, Chuanli Liu, Lingzhong Xu

**Affiliations:** ^1^Centre for Health Management and Policy Research, School of Public Health, Cheeloo College of Medicine, Shandong University, Jinan, China; ^2^National Health Commission Key Laboratory of Health Economics and Policy Research, Cheeloo College of Medicine, Shandong University, Jinan, China; ^3^Center for Health Economics Experiment and Public Policy Research, Shandong University, Jinan, China

**Keywords:** living arrangements, depression, social support, older adults, mediating effect

## Abstract

**Background:**

Living arrangements and social support have an impact on depression among older adults. However, the underlying mechanism between those variables remains unknown. This study aims to investigate the mediating role of social support in the relationship between living arrangements and depression among older adults.

**Materials and methods:**

Multi-stage stratified sampling method was used to select 3,859 older adults from Taian City, Shandong Province, China, for cross-sectional investigation. Living arrangements were measured by a question. Social support and depression were measured using the Multidimensional Scale of Perceived Social Support and Patient Health Questionnaire-9. Multiple linear regression models were used to assess the relationship between living arrangements and depression and the possible influence of social support on the relationship between living arrangements and depression.

**Results:**

Statistics showed that 15.08% of older adults lived alone. After controlling for covariates, living arrangements (ß = 0.45, *t* = 2.87, *P* < 0.01) and social support (ß =−0.08, *t* =−16.93, *P* < 0.001) were significantly associated with depression. The linear regression model showed that social support mediated the relationship between living arrangements and depression, and the mediating effect accounted for 18.20% of the total effect.

**Conclusion:**

This study revealed that living arrangements played an essential role in indirectly predicting depression in older adults through social support. This provided evidence for how to reduce depression in older adults.

## Introduction

Population aging is a major social problem today, and China is no exception. Older adults are proliferating both in quantity and proportion of the total population. Compared with 2010, the proportion of China’s population aged over 60 increased by 5.44% in 2020 (1). It is predicted that 400 million people will be aged 65 and over by 2050, of which 150 million are aged 85 and over (2, 3). Therefore, China faces challenges brought on by the aging population, such as the increased risk of depression in old age, etc. (4, 5).

Depression, the most common mental disorder among the elderly, is one of the aging population’s disease burdens (6). According to statistics, approximately 31.2% of older adults in China suffer from depression symptoms (7). Geriatric depression is a severe disease. Some studies have found that geriatric depression will increase the morbidity and mortality of severe physical disorders and is associated with adverse health outcomes (8, 9). Positive mental health outcomes are an essential criterion for successful aging, directly related to the quality of life and well-being of the elderly (10). Therefore, the depression problem of the elderly should not be ignored. Studies on subjects with depressive symptoms or depression have demonstrated that both were associated with less social support, the condition of living alone, and a high perception of poor health conditions (11, 12). These findings played an important role in exploring the pathogenesis of depression and preventing depression in older adults.

With the development of China’s economy and the change in family structure, more than 50% of older adults living alone in China, and living arrangements have become a social issue that cannot be ignored (13). The severity of geriatric depression might vary dramatically depending on living arrangements (14). Previous studies have shown the relationship between living arrangements and depression, but the conclusions were not consistent (5, 15–17). A cross-sectional study from South Korea reported that living without a spouse in their family and living alone were most strongly associated with depressive symptoms (15). A study from Malaysia demonstrated that older people living with their family members were at a higher risk of developing depression (16), and another meta-analysis study proved that there was no significant association between living arrangements and depressive symptoms (5).

Social support is a person’s perception of the availability of help or support for others in their social networks, including parents, children, spouses, etc. (18). Social support is a vital concept closely related to living arrangements, which might play an essential role in predicting depression among older adults (19). The current studies have shown that older adults who lack of social support were more likely to suffer from depression (20–22). Social support could reduce the risk of depression through the benefits of social relationships and alleviate adverse psychological outcomes (23). Moreover, social support is also related to living arrangements. Older adults’ social support can vary significantly between different living arrangements (24). Previous studies have shown that older adults who live alone may receive less social support than those who live with a spouse or family members (25–27). Additional studies have shown that social support mediated the relationship between loneliness and depression in older adults (28). Even so, living arrangements and loneliness are not identical, it is also possible to feel lonely while living with others. Based on the literature review, it seems that there is a potential mechanism between living arrangements, social support, and depression. Social support may be a potential mediator of the relationship between living arrangements and depression.

Although the direct effects of living arrangements and social support on depression have been widely discussed, to our knowledge, there have been no studies on the existing mechanistic relationship between these three aspects in older adults. Therefore, the aim of this study was to: (1) examine the relationship between living arrangements and depression among Chinese older adults; (2) explore the effect of social support on the relationship between living arrangements and depression in the elderly.

## Materials and methods

### Data collection and subjects

Data were collected from the 2020 Household Health Interview Survey, a cross-sectional survey conducted in Taian City, Shandong Province, China. Multi-stage stratified cluster random sampling method was applied to select participants from all six administrative districts (four counties and two districts). In the first stage, according to the level of economic and social development (per capita GDP level) and geographical location, three or four streets (towns) were randomly investigated in each district (county), and a total of 20 sub districts/towns were selected. In the second stage, eight villages/committees were randomly selected in each township/street, and 160 subdistricts/towns were selected. Finally, 50 households were randomly selected in each village/committee. All participants were interviewed face-to-face by trained interviewers using a structured questionnaire. Among the 7,945 households interviewed, 7,921 completed the questionnaire. A total of 8,542 individuals over 15 were included in this sample. We selected 3,859 older adults aged 60 and over as subjects in this study.

This study was approved by Ethical Committee of the School of Public Health, Shandong University (approval number LL20191220). All participants filled out the informed consent form before the investigation.

### Measurement

#### Assessment of living arrangements

We measured participants’ living arrangements by asking: “How many people have lived in your house in the last 6 months? (including yourself),” and we divided the answers into two categories: living alone and not living alone.

#### Assessment of social support

The Multidimensional Scale of Perceived Social Support (MSPSS), which was developed by Zimet et al. (29), was employed. The MSPSS consisted of 12-item self-reported questions to assess aspects of perceived social support. The subjects were asked to rate each item on a seven-point Likert scale ranging from 1 (very strongly disagree) to 7 (very strongly agree). The range of total scale scores was 12–84; the higher scores indicated more perceived social support (29, 30). We used the Chinese version of MSPSS, and this scale was widely used in China with good reliability and validity (31, 32). The Cronbach’s alpha coefficient for this scale was 0.94.

#### Assessment of depression

Depression was accessed using Patient Health Questionnaire-9 (PHQ-9) (33). The scale was a self-assessment tool for depression based on the Diagnostic and Statistical Manual of Mental Disorders (DSM-IV) in the United States. There were nine items on the scale, and the answers to each item included four choices, which were “none at all,” “occasional days,” “more than half of the days,” “almost every day, and the corresponding scores were 0, 1, 2, 3. The score range is 0–27 points. The higher the score, the higher the likelihood of depression (0–4: no depression, 5–9: mild depression, 10–14: moderate depression, 15–19: moderate to major depression, 20–27: major depression). The scale was widely used in China with good reliability and validity (34, 35). The Cronbach’s alpha coefficient for this scale was 0.84.

#### Measurement of sociodemographic variables

We included the following sociodemographic characteristics as control variables: gender (male or female), age, marital status (married or others), the condition of residence (urban or rural), education level (illiterate, primary school, middle school, high school, and above), employment status (yes or no), smoking (yes or no), drinking (yes or no), self-reported health status (poor, fair, good), the number of chronic diseases (hypertension, diabetes, coronary heart disease, stroke, cerebral thrombosis, osteoarthrosis, and other chronic diseases diagnosed by doctors), and annual household income. Participants were asked about annual household income through written statements. They made their best estimate of total household income to the extent provided, including income from wages or allowances, as well as from unemployment benefits, pensions, investments, the assistance provided to families, or other government or non-government benefits in the previous 12 months. And this variable was divided into low- to high-quartile groups (Q1, Q2, Q3, Q4).

### Statistical analysis

We used descriptive statistics to describe the sample, and independent *t*-test and Chi-square test were performed to compare differences between groups of different sociodemographic characteristics. Spearman correlation analysis was used to verify the correlation between living arrangements, social support, and depression. Since the dependent variable of this study was depression, the hierarchical multiple linear regression method (36) was used to access the mediating effect. The results of the regression model were presented as a beta coefficient and 95% confidence interval (95% CI). Finally, the bootstrap test was employed to verify the mediation effect and draw the mediation effect model. All data were analyzed using SPSS 25.0. *P*-values < 0.05 were considered statistically significant.

## Results

### Demographic characteristics of subjects

A total of 3,859 older adults were included in our study. The mean age of subjects was 68.80 years (standard deviation [SD] = 6.00, range: 60–93 years). There were 3,277 subjects who did not live alone and 582 subjects who lived alone. About 72.10% of them lived in rural areas, and 27.90% lived in urban areas. About 44.20% of the subjects were illiterate or semi-illiterate. About 77.20% of the subjects were married at the time of the interview and 15.08% were living alone. Characteristics associated with older adults living alone included lower social support scores, higher depression scores, being female, rural, lower education level, not in a marriage, being unemployed, smoking, drinking, and lower annual household income (*P* < 0.05) (Table 1).

**TABLE 1:** Sociodemographic characteristics of the participants [Mean ± SD/*n* (%)].

Characteristics	All (*n* = 3,859)	Not living alone (*n* = 3,277)	Living alone (*n* = 582)	*P* ^a^
Social support	66.84 ± 12.03	67.12 ± 11.68	65.22 ± 13.75	0.001
Depression	3.04 ± 3.83	2.90 ± 3.67	3.83 ± 4.54	<0.001
Gender				<0.001
Male	1,568 (40.60%)	1,403 (42.80%)	165 (28.40%)	
Female	2,291 (59.40%)	1,874 (57.20%)	417 (71.60%)	
Age				<0.001
60∼69	2,271 (58.80%)	2,055 (62.70%)	216 (37.10%)	
70∼79	1,370 (35.50%)	1,088 (33.20%)	282 (48.50%)	
≥80	218 (5.60%)	134 (4.10%)	84 (14.40%)	
Residence				0.118
Rural	2,781 (72.10%)	2,346 (71.60%)	435 (74.70%)	
Urban	1,078 (27.90%)	931 (28.40%)	147 (25.30%)	
Education level				<0.001
Illustrate/semi-illustrate	1,707 (44.20%)	1,371 (41.80%)	336 (57.70%)	
Primary school	830 (21.50%)	707 (21.60%)	123 (21.10%)	
Middle school	836 (21.70%)	756 (23.10%)	80 (13.70%)	
High school and above	486 (12.60%)	443 (13.50%)	43 (7.40%)	
Marital status				<0.001
Married	2,981 (77.20%)	2,926 (89.30%)	55 (9.50%)	
Others	878 (22.80%)	351 (10.70%)	527 (90.50%)	
Employment status				<0.001
Yes	1,224 (31.70%)	1,103 (33.70%)	121 (20.80%)	
No	2,635 (68.30%)	2,174 (66.30%)	461 (79.20%)	
Smoking				0.05
Yes	588 (15.20%)	515 (15.70%)	73 (12.50%)	
No	3,271 (84.40%)	2,762 (84.30%)	509 (87.50%)	
Drinking				<0.001
Yes	857 (22.20%)	765 (23.30%)	92 (15.80%)	
No	3,002 (77.80%)	2,512 (76.70%)	490 (84.20%)	
No. of chronic diseases				0.928
None	1,013 (26.30%)	882 (26.90%)	131 (22.50%)	
1	1,226 (31.80%)	1,051 (32.10%)	175 (30.10%)	
≥2	1,620 (42.00%)	1,344 (41.00%)	276 (47.40%)	
Annual household income				<0.001
Q1 (lowest)	1,332 (34.50%)	956 (29.20%)	376 (64.60%)	
Q2	1,684 (43.60%)	1,514 (46.20%)	170 (29.20%)	
Q3	661 (17.10%)	634 (19.30%)	27 (4.60%)	
Q4 (highest)	182 (4.70%)	173 (5.30%)	9 (1.50%)	
Self-reported health status				0.125
Good	2,164 (56.10%)	1,847 (56.40%)	317 (54.50%)	
Fair	1,205 (31.20%)	1,029 (31.40%)	176 (30.20%)	
Poor	490 (12.70%)	401 (12.20%)	89 (15.30%)	

^a^Used independent *t*-test and Chi-square test.

### Correlation between living arrangements, social support, and depression

Spearman correlation analysis was conducted on living arrangements, social support, and depression. As shown in Table 2, living arrangements were negatively correlated with social support (*r_*s*_* = −0.041, *P* < 0.05) and positively associated with depression (*r_*s*_* = 0.069, *P* < 0.01). Social support was negatively associated with depression (*r_*s*_* = −0.294, *P* < 0.01).

**TABLE 2:** Correlation analysis of living arrangements, social support, and depression.

Variables	1	2	3
1. Living arrangements	–		
2. Social support	−0.041*	–	
3. Depression	0.069**	-0.294*	–

***P < 0.001, **P < 0.01, *P < 0.05.

### The mediating effect of social support on living arrangements and depression

There was a significant correlation between living arrangements and depression, which can further analyze the mediating effect among older adults. After controlling for demographic variables (gender, age, residence, education level, employment status, annual household income, the number of chronic diseases and self-rated health status), Baron and Kenny’s (36) hierarchical multiple linear regression method was used to test whether social support had a mediating effect on living arrangements and depression. The variance inflation factors of all independent variables were less than 10, and there was no multicollinearity.

Model 1 included living arrangements and depression, model 2 included living arrangements and social support, and model 3 included living arrangements, social support and depression After adjusting for the covariates in all models, Model 1 showed that living arrangements had a significant direct prediction effect on depression (β = 0.55, *t* = 3.53, *P* < 0.001); Model 2 included living arrangements and social support, and the data showed that living arrangements had a significant direct predictive effect on social support (β = −1.25, *t* = −2.90, *P* < 0.01); In model 3, after adjusting living arrangements, social support had a significant predictive effect on depression (β = −0.08, *t* = −16.93, *P* < 0.001). The predictive effect of living arrangements on depression was still significant, but the predictive effect was weakened (β = 0.45, *t* = 2.87, *P* < 0.01). It indicated that social support mediated the influence of living arrangements on depression (Table 3).

**TABLE 3:** Regression analysis of mediating model of social support.

Variables	Model 1 (Depression)			Model 2 (Social support)			Model 3 (Depression)		
			
	β	*t*	95% CI	β	*t*	95% CI	β	*t*	95% CI
Living arrangements	0.55***	3.53	[0.26, 0.90]	−1.25**	–2.90	[−2.68, −0.52]	0.45**	2.87	[0.14, 0.76]
Social support							−0.08***	–16.93	[−0.09, −0.07]
**Gender (0 = Female)**									
Male	0.57***	4.26	[0.31, 0.83]	0.91	2.04	[0.04, 1.79]	0.64***	4.97	[0.39, 0.89]
**Age (0 = 60∼69)**									
70∼79	–0.23	–1.84	[−0.48, 0.02]	–0.04	–0.10	[−0.88, 0.80]	–0.24	–1.93	[−0.48, 0.01]
≥80	–0.51	–1.97	[−1.02. 0.01]	1.16	1.31	[−0.57, 2.86]	–0.42	–1.69	[−0.91, 0.07]
**Residence (0 = Rural)**									
Urban	–0.19	–1.36	[−0.45, 0.08]	0.34	0.73	[−0.57, 1.24]	–0.16	–1.21	[−0.42, 0.10]
**Education level (0 = Illiterate)**									
Primary school	−0.36**	–2.32	[−0.67, −0.06]	–0.05	–0.10	[−1.08, 0.98]	−0.36*	–2.43	[−0.66, −0.07]
Middle school	−0.56**	–3.37	[−0.88, −0.23]	0.57	1.12	[−0.53,1.66]	−0.51**	–3.21	[−0.83, −0.20]
High school and above	−0.65**	–3.23	[−1.05, −0.26]	0.58	0.86	[−0.75,1.91]	−0.61**	–3.11	[−0.99, −0.22]
**Employment status (0 = Yes)**									
No	0.24	1.80	[−0.02, 0.50]	–0.60	–1.32	[−1.48, 0.29]	0.19	1.50	[−0.06, 0.45]
**Annual household income (0 = Q1)**									
Q2	−0.26*	–2.14	[−0.49, −0.02]	0.73	1.81	[−0.06, 1.51]	–0.20	–1.73	[−0.43, 0.03]
Q3	−3.47***	–18.71	[−3.84, −3.11]	6.10***	9.76	[4.88, 7.33]	−3.00***	–16.52	[−3.35, −2.64]
Q4	–0.44	–1.56	[−0.98, 0.11]	0.67	0.70	[−1.17, 2.50]	–0.38	–1.43	[−0.91, 0.14]
**No. of chronic diseases (0 = None)**									
1	0.42**	2.79	[0.13, 0.72]	–0.56	–1.09	[−1.56, 0.44]	0.38*	2.60	[0.09, 0.67]
≥2	1.23***	8.08	[0.93, 1.53]	–1.23	–2.39	[−2.24, −0.22]	1.14***	7.72	[0.85, 1.43]
**Self-reported health status (0 = Poor)**									
Fair	−2.40***	–12.66	[−2.77, −2.03]	3.46***	5.42	[2.21, 4.71]	−2.13***	–11.59	[−2.49, −1.77]
Good	−3.72***	–20.36	[−3.84, −3.11]	6.10***	9.76	[4.88, 7.33]	−3.00***	–16.52	[−3.35, −2.64]
*R* ^2^	0.17			0.05			0.21		
*F*	52.23***			12.12***			424.09***		

***P < 0.001, **P < 0.01, *P < 0.05; Q: quartile.

**FIGURE 1:**
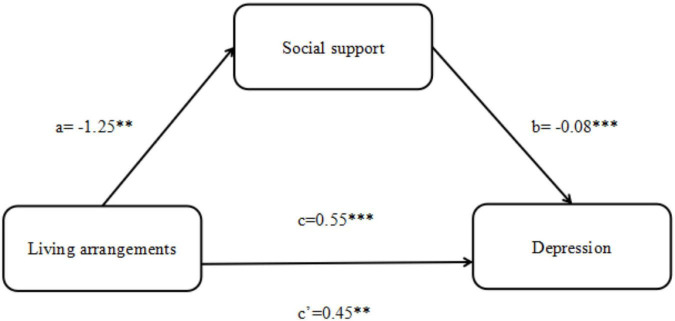
The framework for social support’ mediating role in the relationship between living arrangements and depression. ***P < 0.001, **P < 0.01, *P < 0.05.

According to the test procedure of the mediation effect, we used the living arrangements as the independent variable, depression as the dependent variable, and social support as the mediating variable to verify the significance of the mediating effect. Table 4 revealed that social support mediated living arrangements and depression. In the mediating effect model with social support as the mediator, the direct effect of living arrangements on depression was 0.45 (95% CI = [0.14, 0.77]), and the indirect effect of social support was 0.10 (95% CI = [0.03, 0.20]), and the total effect was 0.55 (95% CI = [0.22, 0.88]). The result indicated that social support partially mediated the relationship between living arrangements and depression, and the mediating effect accounts for 18.20% of the total effect. To more clearly present the results of hypothesis testing between social support and living arrangements and depression, we draw a mediating effect model (Figure 1).

**TABLE 4:** Boostrap analysis of the mediating effects of social support.

Path	β	BootSE	95% CI		Percentage of total effect (%)
			
			Lower	Upper	
Total effect	0.55	0.17	0.22	0.88	100
Direct effect	0.45	0.16	0.14	0.77	81.80
Indirect effect	0.10	0.05	0.03	0.20	18.20

## Discussion

This study examined the relationship between living arrangements and depression among older adults and verified the mediating effect of social support. The results indicated that living arrangements were significantly associated with depression in older adults, and social support mediated the effect of living arrangements on depression.

Living arrangements were significantly associated with depression in older adults. This result was consistent with some studies that confirmed the relationship between living arrangements and depression (37–39). The type of living arrangements was necessary for depression in older adults because it played an influential role in defining social roles, providing social support functions and interactions, and alleviating adverse psychological conditions (40). Older people living alone were more likely to be depressed than those who lived with others. A Japanese cross-sectional study reported that living alone without a partner and spouse was associated with a higher risk of depression (41). Another US cross-sectional study has also indicated that living alone (compared to living with a family member) was associated with elevated levels of depressive symptomatology (42).

We creatively revealed the potential mediating role between living arrangements and depression. As expected, the mediating effect analysis demonstrated that social support mediated the relationship between living arrangements and depression. Consistent with previous studies (21, 43), social support negatively affected depression. One study reported that high perceived social support predicted reduced depression severity (44). Older adults with a higher level of social support were more likely to feel the support and interaction of family and individuals and thus had a lower depression tendency. When the elderly received low social support, they were prone to psychological problems and negative emotions (45). Social support is the critical factor that affects geriatric depression. The elderly’ activity spectrum is relatively limited, and their mode of life is monotonous and boring; increasing the solitary old adults’ family interaction and participation and let them get more emotional support might effectively improve their perceived social support, reduce the risk of depression (46–48). The living arrangements could influence social support and depression and indirectly affect depression by influencing social support in older adults. This finding was supported by earlier studies, such as one that found that the elderly who lived alone had the lowest level of social support and were more likely to suffer from depression or other negative emotions than those who lived with their families (49). It can be understand that older adults who live with others have more interpersonal contacts, closer social relations, higher social support, and lower depression tendency than those who live alone.

The findings had critical practical applications in protecting older people from depression. Due to the mediating role of social support in the relationship between living arrangements and depression, appropriate interventions should be considered for older adults living alone. Social participation and physical activity could buffer stress and combat the onset and development of mental illness (50). Therefore, policymakers can build quality communities to strengthen physical activity and expand social networks for older people living alone. Furthermore, in 2013, the Chinese government enacted a law mandating children to visit their parents. However, millions of older adults who live alone still experience long-term isolation (51). Their depressive symptoms are easily overlooked compared to their physical health, and long-term improvement of depression among older adults living alone needs the joint efforts of the government and families.

One of the strengths of this study was that we surveyed a broad community population, and the results were more accurate and stable. There were also several limitations. Firstly, the data of this study was derived from a cross-sectional study, so the causality cannot be verified. Longitudinal studies can more accurately measure the variation of variables over time and verify the causal relationship between variables. Secondly, we analyze the overall social support scale. We can study the three dimensions of the scale, respectively, in the future and get more targeted results. Thirdly, due to the limited on-site investigation during COVID-19 prevention and control, the samples of this study were only from Taian City, Shandong Province, China. The sample size can be expanded to validate this study further. Furthermore, only two types of living arrangements were included in the study. To improve the study, the variable “not living alone” can be further classified in future studies.

## Conclusion

This study demonstrated that living arrangements had a significant negative predictive effect on depression. Older adults who lived alone were more likely to be depressed than those who lived with others. We also confirmed that living arrangements directly impact depression in the elderly and can also indirectly impact depression through social support. This study provided insights into policy recommendations to improve depressive symptoms among older adults.

## Data availability statement

The raw data supporting the conclusions of this article will be made available by the authors, without undue reservation.

## Ethics statement

The studies involving human participants were reviewed and approved by Ethical Committee of the School of Public Health, Shandong University (approval number: LL20191220). The patients/participants provided their written informed consent to participate in this study.

## Author contributions

ZP, YZ, XZ, XC, and CL conducted the study implementation and data collection to collate and interpret the data. ZP performed the statistical analysis and wrote the manuscript. LX, WQ, and FH helped to revise the manuscript. LX directed the study. All authors made significant contributions to the manuscript, critically reviewed the draft version and provided important revision suggestions for the content, and approved the final manuscript.
